# Electroencephalographic aspects and phenotypic characteristics in children with autism

**DOI:** 10.25122/jml-2025-0053

**Published:** 2025-03

**Authors:** Alexandru Capisizu, Leon Zăgrean, Adriana Sorina Capisizu

**Affiliations:** 1 Dr. Constantin Gorgos Psychiatry Hospital, Bucharest, Romania; 2 Division of Physiology and Neuroscience, Department of Functional Studies, Carol Davila University of Medicine and Pharmacy, Bucharest, Romania; 3 Department of Radiology and Imagistic Medicine 1, Carol Davila University of Medicine and Pharmacy, Bucharest, Romania

**Keywords:** Autism spectrum disorder, electroencephalography, neurological examination, children

## Abstract

Autism is a severe neurodevelopmental disorder that affects many individuals around the world, with a constantly increasing prevalence. The association between autism and electroencephalographic (EEG) abnormalities in children suggests a worse evolution of clinical features. A retrospective study was conducted, including 101 children with autism who underwent clinical and neurological examination and wake electroencephalography. This study aimed to examine EEG abnormalities in children with autism, identify phenotypic characteristics associated with these abnormalities, asses their clinical relevance, and determine potential phenotypic correlations. The results showed that 10.89% of the patients in the study presented EEG abnormalities, similar to those of other studies that used wake EEG. Of these patients, 18.18% presented epileptic-type discharges, such as spike and wave complexes, and 81.81% presented non-epileptic-type abnormalities, such as bursts of slow waves, generalized or focal. Regarding the phenotypic profile of the patients with EEG abnormalities, 45.45% had a positive family history, 63.63% presented with dysmorphic features and 27.27% presented with gait disturbance. This study shows that some children with autism present multiple EEG abnormalities and diverse phenotypic traits in terms of personal and family history, dysmorphic features, and neurological examination. Identifying EEG abnormalities can improve clinical decisions with complex treatment and monitoring of co-occurring conditions like epilepsy. The use of accessible, effective, and noninvasive assessment tools, such as EEG recordings and neurological examinations in children with autism, can provide valuable support for improved case management.

## INTRODUCTION

Autism Spectrum Disorder (ASD) is a group of severe and persistent neurodevelopmental disorders. According to the Diagnostic and Statistical Manual of Mental Disorders, Fifth Edition (DSM-5), published by the American Psychiatric Association, ASD is characterized by a triad of core deficits: impairments in social interaction, deficits in verbal and non-verbal communication, and restricted, repetitive and stereotyped pattern of interests and activities [1]. Recent data from the 2023 Community Report of the Centers for Disease Control and Prevention (CDC) Autism and Developmental Disorders Surveillance Network indicate that the global prevalence of ASD in children is estimated at 2.7% or approximately one in 36 children [2]. This represents an increase from earlier estimates of one in 54 children in 2016 [3] and one in 59 in 2014 [4].

Epilepsy, the most common neurological disorder, affects approximately 50 million individuals worldwide, with prevalence rates varying from 5.8% in developed countries to 12.7% in rural areas of developing countries [5]. The International League Against Epilepsy (ILAE) defines epilepsy as an enduring predisposition to generate epileptic seizures, typically diagnosed after two unprovoked seizures occurring more than 24 hours apart. Epilepsy syndromes are further characterized by specific clinical and electroencephalographic features, often supported by genetic, structural, or metabolic findings [6].

Electroencephalography (EEG) is the most widely used investigation method for recording the bioelectrical activity of the brain [7] and the paraclinical method of choice for the diagnosis of epilepsy, with the role of identifying the type of epilepsy [8] and classifying epileptic syndromes, according to the ILAE [9]. Many researchers have suspected that paroxysmal epileptic discharges, without being conventionally defined as epileptic seizures, may have important neuropsychiatric and neurobehavioral consequences [10]. Recent research indicates that epileptic discharges are not asymptomatic. In children with ASD, abnormal EEG findings may indicate more severe clinical outcomes, with lower adaptative functioning, cognitive impairment, and clinical and neurological deficits [11,12].

This study aimed to examine EEG abnormalities in children with autism and to identify the phenotypic characteristics associated with these abnormalities, including personal and family history, clinical and neurological examination, and neuroleptic treatment, as well as to determine potential correlations.

A significant yet variable prevalence of EEG abnormalities has been documented in non-epileptic children with ASD [13]. The prevalence of EEG abnormalities in patients with autism has been reported to range from 8% to 80% by Bosetti *et al*. [13] and from 4% to 86% by Prenzano *et al*. [14] in subclinical EEG abnormalities. The type of EEG contributes to this variability, with a lower prevalence in studies using wake recordings and a higher prevalence in long-term and sleep EEG recordings [12]. Regarding the type of abnormalities recorded on the EEG, the prevalence of epileptic-type EEG abnormalities in patients with autism alone ranges from 28% [15] to 30% [14], while non-epileptic abnormalities, such as paroxysmal slow wave activity, have been observed in up to 58% of cases [15].

In Romania, limited research has addressed EEG abnormalities in children with autism. Although some studies have evaluated children with autism using the ABAS scale, neurological examinations, and EEG assessments [16], most have focused primarily on clinical symptomatology [17]. Within this context, this study is important to fill a gap in the literature on electroencephalographic abnormalities in children with autism and their phenotypic profile characteristics.

## MATERIAL AND METHODS

### Study design and patients

We performed a retrospective, single-center study on children diagnosed with autism who underwent EEG and neurological examinations at the Dr. Constantin Gorgos Hospital in Bucharest, Romania, between February 2021 and April 2023. The study was approved by the hospital’s ethics committee (no. 4747/02.12.2020) and conducted following the Declaration of Helsinki. Written informed consent was obtained from all patients. Patients were included if they met the predefined inclusion and exclusion criteria.

The inclusion criteria were patients aged between 2 and 18 years, previously diagnosed with autism by their attending pediatric psychiatrist according to the ICD-10 diagnostic criteria [18], those who received a clinical neurological evaluation and a wake EEG examination by a pediatric neurologist, and patients whose EEG recordings showed abnormalities. The exclusion criteria were patients previously diagnosed with epilepsy and those who could not perform the EEG investigation.

A total of 101 children with autism, aged between 2 and 18 years, were evaluated through neurological examination and wake EEG, of whom 11 (10.89%) exhibited EEG abnormalities.

Personal history was considered positive in the presence of prenatal maternal pathology, fetal distress, or any perinatal complications. A positive family history was defined as a documented presence of psychiatric pathology among family members. Dysmorphic features in the clinical examination were considered positive in cases of craniofacial dysmorphisms (e.g., macrocrania, craniosynostosis, or microcephaly), weight and stature abnormalities, anomalies of the limbs, or anomalies of the skin (e.g. cafe-au-lait spots, angiofibromas, or achromic spots). Neurological examination was considered positive for abnormalities in fine motor skills, motility, gait, muscle tone, coordination, osteotendinous reflectivity, and cranial nerves examination.

Medical information related to neuroleptic treatment was collected from the patient’s medical records.

### Methodology

Data regarding patients’ history and clinical, psychiatric, and neurological examinations were retrieved from the medical records. The wake standard EGGs were analyzed for all patients by a pediatric neurologist with 10 years of experience in EEG interpretation.

### Medical investigation – Electroencephalogram (EEG)

Patients underwent a standard wake EEG using 19 cephalic bridge-type electrodes arranged in a bipolar montage, following the international 10-20 system, with a reference system and an EKG line. The recordings lasted between 15 and 20 minutes and were performed in accordance with the International League Against Epilepsy recommendations [19]. The EEG recordings were performed following the next steps: eyes open, eyes closed, hyperventilation, and intermittent light stimulation. EEG results were considered positive if the patient showed epileptic or non-epileptic abnormalities. The following abnormalities were considered epileptic: spikes, polyspikes, spike and wave complexes, or sharp waves. The following abnormalities were considered non-epileptic: abnormal focal or generalized activity producing either a slower or faster pathway [20].

### Statistical analysis

The patient database was constructed and analyzed using IBM SPSS version 23 (IBM, Chicago, IL, USA). Qualitative data were expressed as frequencies and percentages, while quantitative variables were summarized using mean values and standard deviations. Group differences were evaluated using the Kruskal–Wallis test, with statistical significance defined as a *P* value <0.05.

## Results

### Phenotypic characteristics of the patients

Among the patients included in the study, 11 (10.89%) presented EEG abnormalities. Of these, eight (72.72%) were male, and three (27.27%) were female participants, with a mean age of 9.36 (+/- 3.72) years. Regarding the patient history among the patients with EEG abnormalities, two (18.18%) had a positive personal history, and five (45.45%) had a positive family history. During the clinical examination, 7 (63.63%) of these patients presented with dysmorphic features.

**Figure 1:**
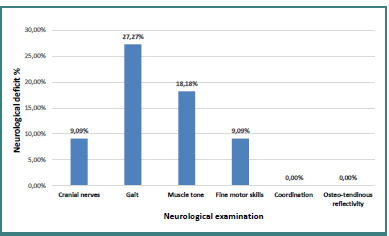
Distribution of patients according to neurological examination

In terms of the neurological examination among the 11 patients with EEG abnormalities, one patient (9.09%) presented with abnormalities of the cranial nerves, three patients (27.27%) presented gait disturbances, two patients (18.18%) had muscle tone anomaly, and one patient (9.09%) had fine motor skills abnormalities. None of the patients presented coordination disorders or osteotendinous reflectivity abnormalities (Figure 1).

Additionally, six (54.54%) of the patients with EEG abnormalities were receiving neuroleptic treatment. Regarding the primary autism diagnosis provided by the pediatric psychiatrist, one (9.09%) patient was diagnosed with childhood autism, eight (72.72%) with atypical autism, and two (18.18%) with other pervasive developmental disorders.

### Electroencephalogram results

**Figure 2:**
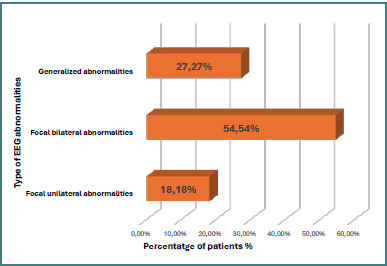
Distribution of patients according to the type of EEG abnormalities

Regarding the EEG records, three patients (27.27%) showed generalized EEG abnormalities, and eight (72.72%) presented focal EEG abnormalities. Among the eight patients with focal EEG abnormalities, two (18.18%) patients showed unilateral focal abnormalities, and six (54.54%) patients had bilateral focal abnormalities (Figure 2).

Among all patients, two (18.18%) presented with epileptic-type abnormalities, and nine (81.81%) of the patients had non-epileptic-type abnormalities. Of the two patients with epileptic-type abnormalities, one had temporo-parietal focal discharges, and the other had generalized discharges. The most representative EEG abnormalities are described below.

### Patient 1

**Figure 3:**
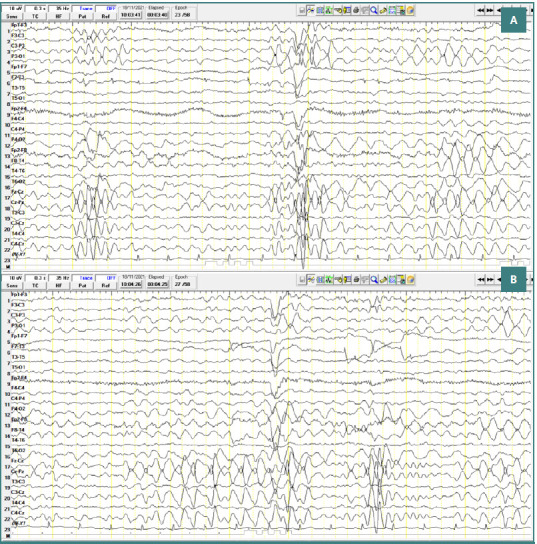
Wake EEG recording. A, Bursts of slow waves with a frequency of approximately 5 Hz and with an amplitude of less than 100 µV, located in the central regions; B, Bursts of slow waves with a frequency of approximately 5 Hz and with a high amplitude of approximately 100-150 µV, located in the central regions.

A 17-year-old male patient diagnosed with childhood autism demonstrated bursts of slow waves with a frequency of approximately 5 Hz. Some bursts had a lower amplitude (less than 100 μV), while others had higher amplitudes ranging from 100 to 150 μV, with variable sharp morphology. These discharges were primarily localized to the central region (Cz-Pz) with occasional bilateral temporo-parietal distribution, repeated throughout the recording, and without clinical correspondence (Figure 3AB).

### Patient 2

**Figure 4:**
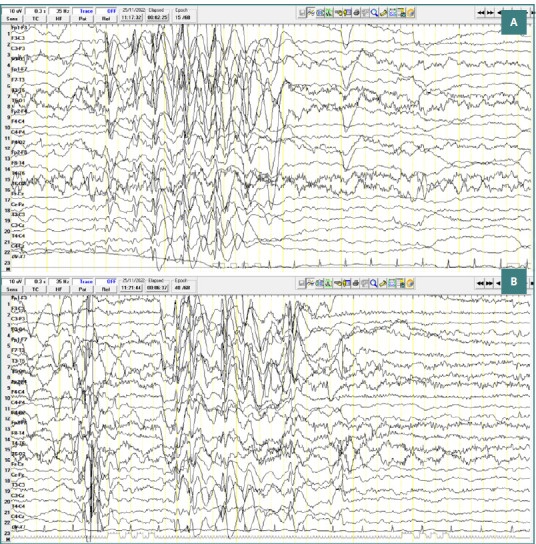
Wake EEG recording. A, Generalized spike-and-wave complexes, with greater amplitude in the left hemisphere, in the central-temporal area, amplitude ~ 400 µV, frequency 3 Hz, duration; B, Generalized spike-and-wave complexes.

A 8-year-old male patient diagnosed with atypical autism presented with two generalized spike-and-wave complexes. These complexes exhibited a higher amplitude in the left hemisphere within the centro-temporal region (corresponding to C3-T3-P3 leads), with an amplitude of approximately 400 µV, a frequency of 3 Hz, and a duration of around 2 seconds. No clinical manifestations were observed during these discharges (Figure 4AB).

### Patient 3

**Figure 5:**
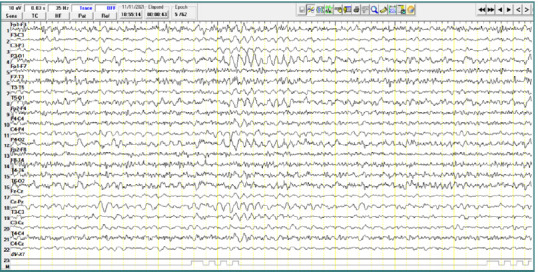
Wake EEG recording. Bursts of slow theta waves with a frequency of 6 Hz, amplitude of approximately 150 µV, with bilateral centro-parietal localization

A 4-year-old male patient diagnosed with atypical autism, presented short bursts of slow theta waves. These waves had a frequency of 6 Hz and an amplitude of approximately 150 µV, with bilateral central-parietal localization, also without any clinically corresponding manifestations (Figure 5).

### Patient 4

**Figure 6:**
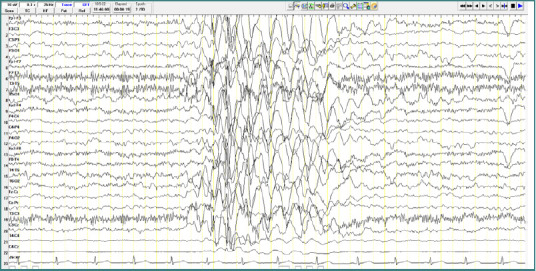
Wake EEG recording. Generalized spike-and-wave complex discharges cluster with an amplitude of approximately 100-150 µV, frequency of 3 Hz, in a display resolution of 10 µV/mm

A 6-year-old female patient diagnosed with atypical autism presented a single generalized spike-and-wave complex discharge cluster with an amplitude of approximately 100-150 µV, a frequency of 3 Hz, and a duration of 2 seconds, without any clinical corresponding manifestations (Figure 6).

### Patient 5

**Figure 7:**
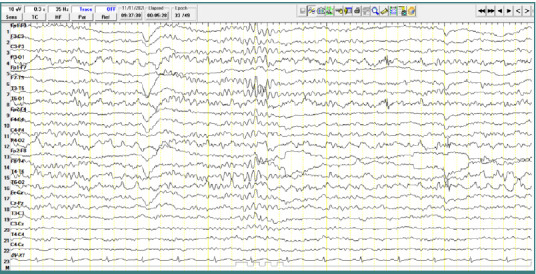
Wake EEG recording. Bilateral focal slow wave bursts, with a frequency of approximately 6-8 Hz, an amplitude of 100 µV, a temporal bilateral localization, and a higher amplitude on the left hemisphere

A 12-year-old male patient diagnosed with atypical autism presented with bilateral focal slow wave bursts with a frequency of approximately 6-8 Hz, an amplitude of 100 µV, a temporal bilateral localization, and a higher amplitude on the left hemisphere, a duration of approximately 1 second, without any clinical corresponding manifestations (Figure 7).

### Patient 6

**Figure 8:**
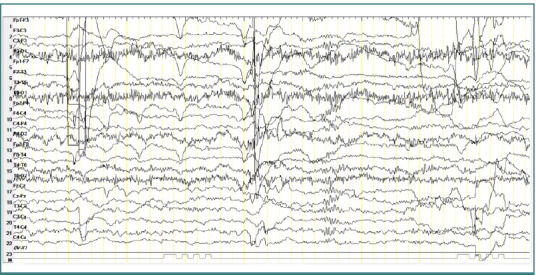
Wake EEG recording. Generalized wave bursts, with a frequency of approximately 6-8 Hz and an amplitude of 100 µV

A 6-year-old male patient diagnosed with atypical autism presented with generalized wave bursts with a frequency of approximately 6-8 Hz, an amplitude of 100 µV, and a duration of approximately 1 second, without any clinical corresponding manifestations (Figure 8).

### Patient 7

**Figure 9:**
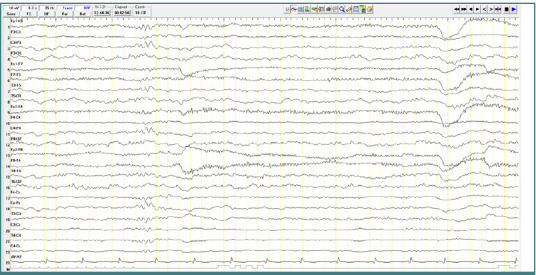
Wake EEG recording. Bilateral focal theta wave bursts, amplitude approximately 20-100 µV, in the centro-parietal area, with a greater amplitude in the left hemisphere

A 13-year-old male patient diagnosed with atypical autism presented bilateral focal theta wave bursts, amplitude approximately 20-100 µV, in the central-parietal area, with greater amplitude in the left hemisphere, without any clinical corresponding manifestations (Figure 9).

### Patient 8

**Figure 10:**
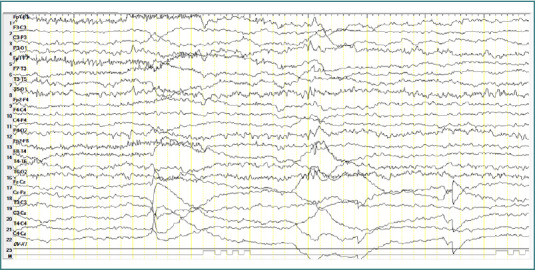
Wake EEG recording. Focal bilateral slow wave bursts, with a frequency of approximately 7 Hz, in the frontal-central-parietal areas of both hemispheres

A 12-year-old male patient diagnosed with atypical autism presented focal bilateral slow wave bursts, with a frequency of approximately 7 Hz, in the frontal-central-parietal areas of both hemispheres without any clinical corresponding manifestations (Figure 10).

### Patient 9

**Figure 11:**
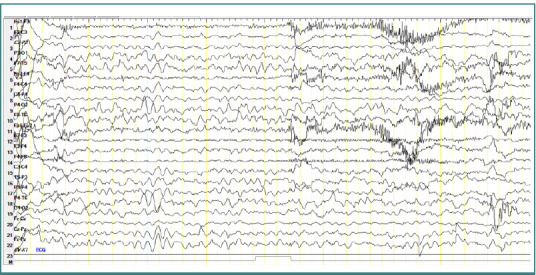
Wake EEG recording. Focal unilateral delta wave bursts, with an amplitude of 200-300 µV, and with right central posterior localization

A 7-year-old female patient diagnosed with atypical autism presented unilateral focal delta wave bursts, with an amplitude of 200-300 µV, with right central posterior localization, without any clinical corresponding manifestations (Figure 11).

### Patient 10

**Figure 12:**
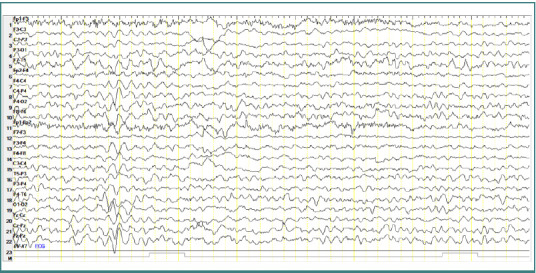
Wake EEG recording. Focal unilateral delta wave bursts, with an amplitude of 200-300 µV, and with right central posterior bilateral localization

A 7-year-old female patient diagnosed with atypical autism presented unilateral focal delta wave bursts, with an amplitude of 200-300 µV, with right central posterior unilateral localization, without any clinical corresponding manifestations (Figure 12). Patient 9 and Patient 10 are twin sisters.

### Patient 11

**Figure 13:**
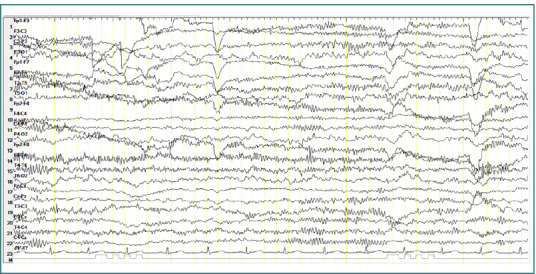
Wake EEG recording. Bilateral focal wave bursts, with an amplitude of 50 µV and a frontal-central bilateral localization

A 11-year-old male patient diagnosed with other pervasive developmental disorders presented bilateral focal wave bursts with an amplitude of 50 µV and a frontal-central bilateral localization without any clinical corresponding manifestations (Figure 13).

### Correlations between EEG abnormalities and the phenotypic profile

**Figure 14:**
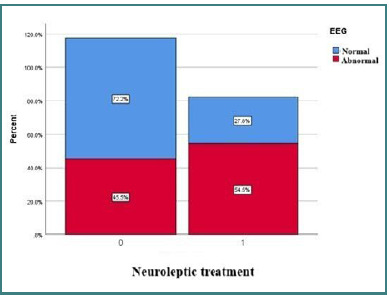
Difference between EEG types depending on neuroleptic treatment

Several correlations were observed between EEG abnormalities and elements of the phenotypic profile. In the overall study population, dysmorphic features were identified in 38 (37.6%) patients, while 31 (30.7%) were receiving neuroleptic treatment. There were borderline statistically significant differences for neuroleptic treatment (*P* = 0.0878) between the group with normal EEG (27.8%) and those with abnormalities (54.5%) [16] (Figure 14).

**Figure 15:**
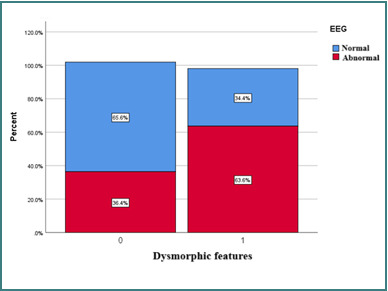
Difference between EEG types depending on dysmorphic features

Also, there were borderline statistically significant differences for dysmorphic features (*P* = 0.0961) between the group with normal EEG (34.4%) and those with abnormalities (63.6%) [16] (Figure 15).

## Discussion

Electroencephalography, due to its non-invasive and safe characteristics, is used in a variety of clinical situations: disease follow-up, monitoring response to treatment, changes in seizure semiology in epileptic patients, management of critically ill patients, comatose patients, patients suspected of status epilepticus or encephalopathy, or in presurgical evaluation [21]. Other specific indications for the pediatric population include genetic syndromes, metabolic disorders, psychomotor regression, and developmental disorders [21].

The routine wake electroencephalography, which contains activation procedures, corresponds to the basic level of EEG recording [19]. The main objective of the standard wake EEG in the diagnosis of epilepsy is the recording of epileptic abnormalities, specifically interictal epileptic discharges. The sensitivity of routine wake EEG may vary depending on age, level of consciousness, sleep deprivation, type of epilepsy, seizure frequency, and antiepileptic treatment [21].

The prevalence of EEG abnormalities in autism tends to be lower in studies using wake recordings. For example, Romero-González *et al*. reported a prevalence of 42%, while other studies have found rates as low as 10.3% in children with ASD [11,16]. Similarly, in the present study, 11 patients (10.89%) exhibited EEG abnormalities; among these, two (18.18%) displayed epileptic-type abnormalities and nine (81.81%) showed non-epileptic abnormalities.

In contrast, studies employing sleep recordings report a higher prevalence of EEG abnormalities. Santarone *et al*., for instance, documented a prevalence of 78% in a group of preschool children with autism [15]. As noted by Capal *et al*., long-term and sleep EEG recordings are associated with a higher proportion of abnormalities [12]. In their study, Santarone *et al*. reported epileptic-like discharges during sleep in 28.4% of subjects. Also, Precenzano *et al*. reported a rate of epileptic discharges in children with autism of 30% during sleep [14,16]. Regarding the prevalence of epileptic-type EEG abnormalities alone in patients with autism, it varies between 28% [15] and 30% [14]. Santarone *et al*. reported non-epileptic abnormalities consisting of paroxysmal slow-wave activity in 58% of subjects, paroxysmal fast-wave activity in 23% of subjects, and asymmetry in 21% of subjects [15,16].

A 2015 review by Ghacibeh *et al*. [22] classified the types of EEG abnormalities in subjects with ASD, including slow rhythm bursts, both generalized and focal, epileptic activity, and epileptic seizures. Epileptic discharges (generalized, focal, and multi-focal) were much more common than non-epileptic EEG abnormalities, with a variety of discharge types. In this study, of all patients with abnormal EEG, three (27.27%) showed generalized EEG abnormalities, and eight (72.72%) presented focal EEG abnormalities. From this study group, five (45.45%) had a positive family history, seven (63.63%) patients presented with dysmorphic features, and regarding the neurological examination, three patients (27.27%) presented with gait disturbance. These results agree with other studies in the literature, such as Miller *et al*., who demonstrated that motor difficulties are a prominent feature of the autism phenotype and require further consideration in the diagnostic criteria [23,24]. Regarding the complexity of autism spectrum disorder, Núñez-Contreras and colleagues published a review in 2022 of the main brain mechanisms underlying the association between autism and epilepsy. Previous observations indicated that there was a higher incidence of epilepsy and autism in preschool and school age, a period in which synaptogenesis and synaptic plasticity are more active. The brain processes that occur during this period are critical for learning, language, and memory; therefore, a disruption of the normal process of synaptic development can lead to the emergence of both types of pathology by abnormalities in glutamatergic and GABAergic systems, which can produce an imbalance between excitatory and inhibitory networks. In conclusion, the author believes the coexistence of autism and epilepsy is an association, not a causal relationship [16,25]. Other authors, such as Keller and collaborators, have also considered that the alteration of proteins involved in neuronal excitability, followed by the limitation of synaptic transmission and the impairment of neuronal function, would play a role in the association of ASD–epilepsy [26].

There are several limitations to this study. First, the study relied on wake EEG recordings, following the practical standards of the clinic, as sleep or long-term EEG are mainly used in cases where there is suspicion of epileptic seizures. Second, the absence of a control group limits our ability to determine whether the EEG abnormalities observed are specific to autism. Other limitations in terms of generalizability were related to the number of patients, mainly since there was a small group of children with autism exhibiting EEG abnormalities and because of its single-center design. Future research should focus on larger, multicentric study groups to validate and extend these findings.

## CONCLUSION

This study showed that a subset of children with autism present multiple EEG abnormalities and diverse phenotypic traits, including variations in personal and family history, dysmorphic features, and neurological examination findings. Identifying EEG abnormalities can improve clinical decisions with complex treatment and monitoring of co-occurring conditions like epilepsy. Future research should incorporate larger sample sizes and include sleep EEG recordings to better identify the EEG abnormalities that most accurately predict treatment outcomes. The use of accessible, effective, and noninvasive assessment tools, such as EEG recordings and neurological examinations in children with autism, can provide valuable support for improved case management.

## Data Availability

Further data are available from the corresponding author upon reasonable request.
